# Silver Nanocoating of LiNi_0.8_Co_0.1_Mn_0.1_O_2_ Cathode Material for Lithium-Ion Batteries

**DOI:** 10.3390/mi14050907

**Published:** 2023-04-23

**Authors:** Xintong Li, Kai Chang, Somia M. Abbas, Rasha S. El-Tawil, Ashraf E. Abdel-Ghany, Ahmed M. Hashem, Hua Wang, Amanda L. Coughlin, Shixiong Zhang, Alain Mauger, Likun Zhu, Christian M. Julien

**Affiliations:** 1 Department of Mechanical and Energy Engineering, Indiana University-Purdue University Indianapolis, Indianapolis, IN 46202, USA; 2 National Research Centre, Inorganic Chemistry Department, Behoes Street, Dokki, Giza 12622, Egypt; 3 Department of Physics, Indiana University, Bloomington, IN 47405, USA; 4 Quantum Science and Engineering Center, Indiana University, Bloomington, IN 47405, USA; 5 Institut de Minéralogie, de Physique des Matériaux et Cosmologie (IMPMC), Sorbonne Université, UMR-CNRS 7590, 4 Place Jussieu, 75752 Paris, France

**Keywords:** lithium-ion batteries, cathode, Ni-rich oxide, surface coating

## Abstract

Surface coating has become an effective approach to improve the electrochemical performance of Ni-rich cathode materials. In this study, we investigated the nature of an Ag coating layer and its effect on electrochemical properties of the LiNi_0.8_Co_0.1_Mn_0.1_O_2_ (NCM811) cathode material, which was synthesized using 3 mol.% of silver nanoparticles by a facile, cost-effective, scalable and convenient method. We conducted structural analyses using X-ray diffraction, Raman spectroscopy, and X-ray photoelectron spectroscopy, which revealed that the Ag nanoparticle coating did not affect the layered structure of NCM811. The Ag-coated sample had less cation mixing compared to the pristine NMC811, which could be attributed to the surface protection of Ag coating from air contamination. The Ag-coated NCM811 exhibited better kinetics than the pristine one, which is attributed to the higher electronic conductivity and better layered structure provided by the Ag nanoparticle coating. The Ag-coated NCM811 delivered a discharge capacity of 185 mAh·g^−1^ at the first cycle and 120 mAh·g^−1^ at the 100th cycle, respectively, which is better than the pristine NMC811.

## 1. Introduction

Rechargeable lithium-ion batteries (LIBs) have found widespread use in portable electronic devices, electric vehicles, and large-scale energy storage systems due to their advantages of high working voltage, high energy density, high-rate capability, and long cycle life [1]. Recently, Ni-rich layered oxides, LiNi_0.8_Co_0.1_Mn_0.1_O_2_ (denoted as NCM811) in particular, have emerged as promising cathode materials for LIBs due to their high capacity, high operating voltage, and low cost [2,3]. However, the high nickel content also causes some technical challenges, such as low-rate capability, substantial capacity loss upon cycling, thermal and structural instability, safety issues, and unstable surface chemical properties [4]. These technical problems may be attributed to several mechanisms. For instance, the irreversible migration of nickel ions to lithium sites results in cation mixing, as in their similar ionic radii, i.e., 0.69 Å vs. 0.76 Å, which may change the layered structure into a spinel and rock salt structure [5]. Residual lithium on the material’s surface can react with water and carbon dioxide in the air to generate a Li_2_CO_3_/LiOH passivation layer, resulting in reaction interface failure of the layered Ni-rich cathodes [6]. Tetravalent nickel ions, oxygen vacancies, and newly generated active oxygen species on the surface of Ni-rich materials are examples of strong oxidizing species that promote the surface side reaction during cycling, leading to the dissolution of transition metals, an increase in interfacial resistance, and eventually, capacity fading [7]. In addition, the unstable delithiated Ni-rich materials could be further dissolved by HF from the side reactions between LiOH and LiPF_6_ to create solid electrolyte interphase (SEI) layer that impedes Li^+^ diffusion [8]. The aforementioned major problems have hindered the practical use of Ni-rich cathode materials in LIBs. To alleviate these drawbacks, various modification techniques have been explored to improve the electrochemical performance of Ni-rich cathode materials, such as coating [9], doping [10], core–shell [11], concentration gradient structures [12], and single-crystalline materials [13]. Among these modifications, surface coating can efficiently remove residual lithium and prevent surface side reactions, stabilizing the surface structure and bulk structure. The coating layer works as an isolation layer to stop side reactions and avoid direct contact between the electrolyte and cathode [14]. Various substances are used to modify the surface structure of active particles, such as electrochemically inert metal oxides with low electronic conductivity [15,16], metal fluorides AlF_3_ [17], metal phosphates [18], glasses [19], lithium-ion conductors [20] and conductive polymers [21]. All these coating materials can also function as HF scavengers, which enhances cycling performance and inhibits impedance growth during cycling [22]. Coating can thus not only suppress side reaction but also improve the electrical contact between electrode particles, contributing to the battery structure optimization and design.

Noble metals, i.e., Ag and Au, have also been utilized as surface modifiers to enhance the performance of electrode materials for LIBs [23,24,25,26,27,28]. Ag is known for its stable chemical and physical properties, including strong thermal conductivity, electrical conductivity, and rich ductility for accelerating electron transfer [29]. Moreover, the cathode material can be protected by Ag coating layer that is less reactive with HF, substituting hydrogen [30]. In this study, Ag-coated LiNi_0.8_Co_0.1_Mn_0.1_O_2_ cathode material coated with silver (Ag-NCM811) is synthesized, using a facile, cost-effective, scalable, and convenient method with 3 mol.% of silver nitrate. We investigated the nature of this Ag coating layer and demonstrated that several phases exist in the deposit, improving the electrochemical properties of the NCM811. To do so, we conducted various characterizations, such as Rietveld refinements of X-ray diffraction (XRD), Raman scattering spectroscopy, X-ray photoelectron spectroscopy (XPS), scanning electron microscopy (SEM), high-resolution transmission electron microscopy (HRTEM) and electrochemical tests using cyclic voltammetry (CV) and galvanostatic charge-discharge (GCD) measurements. The Ag-coated NCM811 demonstrated promising electrochemical results, with higher initial capacity and better capacity retention than the pristine NCM811.

## 2. Materials and Methods

### 2.1. Synthesis

The pristine NCM811 sample was synthesized using the sol–gel method with ethylenediaminetetraacetic acid (EDTA) as a chelating agent. Stoichiometric amounts of metal acetates (CH_3_COOLi·2H_2_O, Ni(CH_3_COO)_2_·4H_2_O, Co(CH_3_COO)_2_·4H_2_O, and Mn(CH_3_COO)_2_·4H_2_O (analytical grade, 99.99%, Sigma-Aldrich, Schnelldorf, Germany) were used as starting materials for the pristine sample with a molar ratio Ni:Co:Mn = 8:1:1. The stoichiometric amounts were mixed together and dissolved in deionized water by stirring for 1 h. To add the EDTA chelating agent safely and gradually into the stirred aqueous solution of all the metal cations at a well-defined pH concentration and temperature, the molar ratio of EDTA to the total metal ions was first adjusted to unity. The pH of the solution was adjusted at ~7 using an alkaline solution of ammonium hydroxide, and the temperature was set at 80 °C. The prepared solution was stirred using magnetic stirring to evaporate until a transparent gel was obtained. The gel was then dried in vacuum. The resultant precursor was heated for 5 h at 450 °C, then ground and recalcined for 10 h at 750 °C with intermittent grinding. To modify the surface of the NCM811 sample, an in situ coating technique was employed using a 3 mol.% ethanolic silver nitrate solution, resulting in the formation of Ag-NCM811. The NCM811 powders and AgNO_3_ were separately dissolved in absolute alcohol. The calculated quantity of AgNO_3_ was added dropwise to the NCM811 suspension. After the mixture was agitated for 1 h, ascorbic acid was used as the reducing agent to reduce Ag^+^ ions to metallic Ag, as shown in Equation (1):

C_6_H_8_O_6_ + 2AgNO_3_ → 2Ag + 2 HNO_3_ + C_6_H_6_O_6_. (1)

An obvious “silver-mirror-like” was observed on the inner wall of vessel after the mixture was continuous stirred for 30 min, indicating the formation of Ag. The resulting mixture was then filtrated using fine filter paper and washed with ethanol. The resulting precursor was subsequently calcined under vacuum at 300 °C for 3 h. Sample synthesis are schematically presented in Figure S1.

### 2.2. Material Characterizations

The crystal structure of the final product was determined using X-ray diffraction (XRD) with a Philips X’Pert apparatus equipped with a CuK_α_ X-ray source (λ = 1.54056 Å) in the 2*θ* range of 10–80°. The morphology of the materials was studied using scanning electron microscopy (SEM) with a JSM-7800F microscope, JEOL, Tokyo, Japan and high-resolution transmission electron microscopy (HRTEM) using a JEOL 2100F microscope operated at 200 kV. X-ray photoelectron spectra were recorded using a scanning X-ray microprobe system (PHI VersaProbe II, Ulvac-Phi, Chanhassen, MN, USA) equipped with a Mg Kα source (λ = 1253.6 eV). Raman spectra were recorded at room temperature with a micro-Raman spectrometer (Renishaw, Wottonunder-Edge, UK) equipped with a confocal Raman microscope in-Via TM system at a 532 nm laser-line excitation. The spectra were calibrated with the reference Si phonon peak at 520 cm^−1^. Brunauer–Emmett–Teller (BET) specific surface area and pore size distribution of synthesized samples were determined from N_2_-adsorption experiments using a Belsorp max version 2.3.2 analyzer (Microstac Retsch GmbH, Haan, Germany).

### 2.3. Electrochemical Measurements

The positive electrodes of lithium cells were prepared by mixing the active materials (NCM811 or Ag-NCM811), carbon black (CB), and polyvinylidene fluoride binder (PVDF) in a mass ratio of 8:1:1. The mixture was added to N-methyl-2-pyrrolidone (NMP) solvent, and stirred magnetically for 24 h to form a homogeneous blend. The well-blended slurry was then cast on an aluminum foil by a doctor blade and dried under vacuum at 100 °C for 24 h. Finally, the electrodes were punched out as discs with a diameter of approximately 11 mm and areal surface of 0.95 cm^2^. CR2032 coin cells were processed in an argon-filled glove box using 30 µL electrolyte, i.e., 1.2 mol L^−1^ LiPF_6_ in ethylene carbonate:ethylmethyl carbonate (EC:EMC) (3:7 by weight) dripped onto the electrode and Celgard 2400 separator. Electrochemical tests were conducted using an Arbin BT2000 battery cycler at room temperature. Prior to cycling, cells were rested for 30 min. Galvanostatic cycling was performed at C/10 and 1 C rates in the voltage range of 2.7–4.3 V vs. Li^+^/Li. Cyclic voltammetry was conducted at room temperature on a BioLogic VSP workstation (Biologic Sci. Instr., Knoxville, TN, USA), with the potential set to sweep from open-circuit voltage to 4.3 V and then back to 2.7 V at a scanning rate of 0.02 mV s^−1^. EIS measurements were conducted on a CHI-680C electrochemical workstation (CH Instruments Inc., Austin, TX, USA) at the spontaneously established open circuit potential (OCP) with an amplitude of 5 mV in a frequency range of 10^5^ to 10^−1^ Hz. The tested impedance data were analyzed using the complex nonlinear least squares fitting (NLSF, ZView software version 2.0) method.

## 3. Results

### 3.1. X-ray Diffraction Analysis

**Figure 1:**
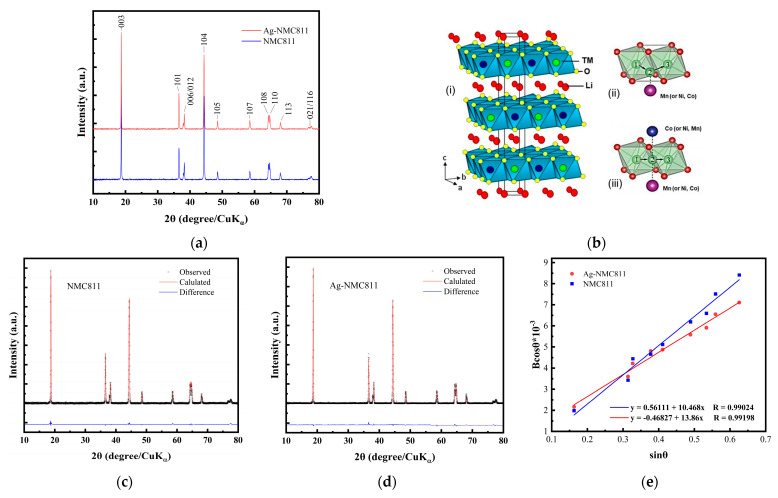
(**a**) XRD patterns of NCM811 and Ag-NCM811 samples recorded with a CuK_α_ X-ray source. (**b**) Scheme of the crystal structure of NCM811. (**i**) Layered *R*$\bar{3}$*m* structure. Colors of atoms are green (Li), red (O), and silver/purple/blue are Ni, Mn, Co transition metal cations, respectively. (**ii**) Tetrahedral site pathway. (**iii**) Oxygen dumbbell pathway for Li^+^ ions. (**c**,**d**) Rietveld refinements of XRD pattern of pristine NCM811 and Ag-NCM811. (**e**) Determination of the microstrain ε from the full-width at half-maximum, *B*_hkl_, of XRD peaks according to Equation (2).

Figure 1a displays the XRD patterns of the as-synthesized NCM811 and Ag-NCM811 samples. No significant difference can be observed, suggesting that the NCM811 particles remain undamaged after silver coating. This indicates that Ag coating does not have much impact on the internal stress for the materials. Both samples exhibit well-resolved reflections with very smooth background, indicating the high crystallinity of NCM811 materials prepared by the sol–gel method without any residual impurities or secondary phases. Therefore, the similarity between the diffractograms of the two samples indicates that silver used in this study does not act as a dopant or intercalant in the structure.

The Bragg lines of both pristine NCM811 and Ag-NCM811 are indexed using the α-NaFeO_2_ layered structure (*R*$\bar{3}$*m* lattice space group, JCPDS card No. 82-1495). The schematic crystal structure of LiNi_0.8_Co_0.1_Mn_0.1_O_2_ is depicted in Figure 1b. As shown in the enlarged diffractograms (Figure S2a–c), the well splitting of (006)/(102) and (108)/(110) pairs reveal a well-ordered layered structure. The intensity ratio of *I*_(003)_/*I*_(104)_ and the *c*/*a* lattice parameter ratio [31] can identify the degree of cation mixing between Ni^2+^ ions and Li^+^ ions. Thus, *c*/*a* ≃ 4.94 and *I*_003_/*I*_104_ ≃ 1.30 are good indicators for a lower degree of cation mixing and better hexagonal ordering of the NCM811 structure. Less cation mixing and structural ordering are beneficial for the electrochemical properties of the host lattice. A careful analysis of the XRD spectra indicates that the (00*l*) reflections of Ag-NCM811 sample are substantially unchanged, confirming that silver has not penetrated inside the layered lattice and remains at the surface without disturbing the main structure. Note that cubic Ag cannot be detected not only because of the low mole percentage loading (~3%), but also due to the overlap of X-ray reflections from native silver with those from NCM811 (see Figure S3). The absence of extra XRD reflections could be attributed to either the formation of an amorphous Ag layer [27] or to the deposit of an amorphous silver oxide that could be AgO, Ag_4_O_4_ or Ag_2_O [24,32]. This hypothesis is supported by the subsequent experiments. Further analysis of the XRD patterns for both samples was performed by Rietveld refinements (Figure 1c,d), and the results are listed in Table 1. In these figures, the cross marks (in black) are experimental data and the solid lines (in red) are the calculated spectra.

The small difference between calculated and experimental diffractograms indicates the high quality of the fitting processes, i.e., refinement parameters *R_wp_* ≈ 10% and *χ*^2^ < 1.4, and validates the structural model. In the Rietveld refinement, the phase fraction was calculated with an uncertainty of 0.1% and evaluated by minimizing the difference between experimental and calculated diffractogram. For Ag-NCM811, the best Rietveld fit showing a good agreement between calculated and observed patterns is obtained by considering that the additional silver-based coating is composed of 1.7 mol.% metallic Ag (Ag^0^, cubic structure, space group *Fm*3*m*) and ~1.6 mol.% silver oxide AgO (Ag^+^) also known as Ag_4_O_4_ (monoclinic structure, space group *P*12_1_/*c*1). This concentration of Ag was confirmed by chemical analysis using inductive coupled plasma (ICP), which showed ~3.3% Ag. Values of the lattice parameters *a* and *c* for NCM811 pristine do not vary significantly upon Ag coating demonstrating the stability of the layered structure. This means that Ag remains at the surface of NCM811 and does not penetrate inside the core of particles. This could be due to the fact that the ionic radius of Ag^+^ (*r*_(Ag^+^)_ = 1.26 Å) is much bigger than that of transition metal ions Ni^3+^ (*r*_(Ni^3+^)_ = 0.56 Å), Ni^2+^ (*r*_(Ni^2+^)_ = 0.69 Å), Co^3+^ (*r*_(Co^3+^)_ = 0.545 Å) and Mn^4+^ (*r*_(Mn^4+^)_ = 0.53 Å) and even Li (*r*_(Li^+^)_ = 0.76 Å). Thus, Ag ions are not hosted by the layered lattice of NCM811, as previously reported [27]. It is worth noting that the value of the *c* lattice parameter of Ag-NCM811 is slightly smaller than that of NCM811, which may be attributed to the lower of Ni^2+^ content, resulting in a decrease in the cation mixing of this sample (Table 1).

The coherence lengths (*L*_c_) values determined from the Scherrer’s formula are 55 ± 2 and 50 ± 2 nm for NCM811 and Ag–NCM811, respectively. Both samples have almost the same crystallite size, which indicates that NCM811 framework is well preserved after coating by Ag. The microstrain (ε) of the NCM811 particles was determined using the Williamson–Hall equation [33]:

*B*_hkl_ cos *θ*_hkl_ = (*K*λ/*L*_c_) + 4 ε sin *θ*_hkl_, (2)

where *B*_hkl_ is the line broadening of a Bragg reflection (hkl), *K* is the shape factor, *L*_c_ is the effective crystallite size and λ is the X-ray wavelength. The microstrain ε is estimated from the slope of the plot *B*_hkl_ cos *θ*_hkl_ vs. 4 sin *θ*_hkl,_ and the intersection with the vertical axis provides the crystallite size (Figure 1e). *B*_hkl_ value used here is the instrumental-corrected one. The values of microstrain are determined to be 0.86 × 10^−3^ and 0.65 × 10^−3^ rd for NCM811 and Ag-NCM811, respectively, indicating a slight difference in crystallinity of the samples, with the microstrain decreased in the presence of Ag coating.

### 3.2. XPS Analysis

**Figure 2:**
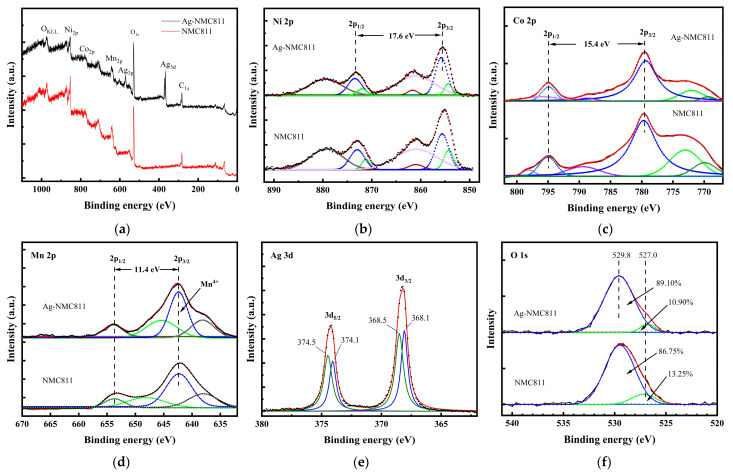
XPS analysis of pristine and Ag-coated NCM811 samples. Survey spectra (**a**). High-resolution XPS spectra of Ni 2p (**b**), Co 2p (**c**), Mn 2p (**d**), Ag 3d (**e**) and O 1s (**f**).

The surface elemental composition and valence state of samples are analyzed by X-ray photoelectron spectroscopy. Figure 2 shows the XPS survey spectrum and high-resolution XPS (HR-XPS) patterns of the Ni 2p, Co 2p, Mn 2p, O 1s, and Ag 3d core levels for the NCM811 and Ag-NCM811 samples. The binding energies (BE) obtained in the XPS analysis were corrected for specimen charging by referencing the C1s line to 284.60 eV. The survey spectrum (Figure 2a) clearly indicates similar binding energies for all species in both samples, which indicating that the chemical states of the transition metal elements in the layered NCM811 structure did not change upon coating with Ag. The Ni 2p spectrum (Figure 2b) shows that the Ni 2p_3/2_ peak appearing at BE of 855.16 eV for pristine NCM811 is slightly shifted towards higher binding energy (855.55 eV) for Ag-NCM811, which can be attributed to a decreased Ni^2+^/Ni^3+^ ratio [34]. This result reveals that more Ni^3+^ ions appear on the Ag-NCM811 electrode surface than in pristine NCM811. The binding energy at BE of 854.29 and 855.46 eV is assigned to Ni^2+^ and Ni^3+^, respectively (Figure 2b). The calculated ratio of Ni^2+^/Ni^3+^ for NCM811 and Ag-NCM811 are 0.308 and 0.263, respectively, which confirms the low degree of cation mixing for Ag-NCM811 in good agreement with XRD data.

Figure 2c presents the HR-XPS spectra of Co 2p core levels, in which the peaks located at BE of 779.6 and 794.9 eV are attributed to the Co 2p_3/2_ and Co 2p_1/2_ orbitals, respectively, with an energy separation Δ*E*_b_ = 15.4 eV indicating the presence of Co^3+^ ions. The HR-XPS spectra of Mn 2p core levels (Figure 2d) evidences the characteristic peaks of Mn 2p_3/2_ and Mn 2p_1/2_ orbitals located at 642.3 and 653.7 eV (with Δ*E*_b_ = 11.4 eV), which suggests that Mn exist in both samples as +4 valence state [35,36]. Figure 2e shows the high-resolution XPS spectrum of Ag 3d_5/2_ and Ag 3d_3/2_ orbitals. By peak differentiating method, four peaks are located at BE of 368.1, 368.5, 374.0 and 374.5 eV. The peaks at 368.1 and 374.0 eV with Δ*E*_b_ = 5.9 eV reveal the presence of Ag^+^ ions (coming from Ag_2_O or Ag^(I,III)^O), while the peaks at 368.5 and 374.5 eV are attributed to metallic Ag^0^ [37,38]. These results indicate that both native Ag and Ag oxide are existent at the surface of Ag-coated NCM811 particles. BE of the 3d_5/2_ core level should be expected to shift towards a higher value; however, the opposite was observed here, indicating the presence of Ag^0^ nanoparticles embedded in a silver oxide matrix instead of a continuous Ag surface, as previously reported [39].

In both materials, the HR-XPS spectrum of O 1s core level (Figure 2f) contains a predominant peak located at 529.8 eV, which is attributed to the transition metal–oxygen (*M*-O) bonds. An additional peak at 527 eV is assigned to the residual oxygen related to impurities with OH− or O− bonding on the surface. Upon comparing the O 1s peaks of NCM811 and Ag-NCM811, the latter sample exhibits a relatively higher content of the lattice oxygen, which reveals a reduction in surface defects after coating. As shown in Figure 2f, the analysis of the O 1s peak reveals the amount of lattice oxygen and surface defect oxygen as reported in Table S1.

The HR-XPS diagrams of NCM811 samples shown in Figure S4, the C 1s core level spectra can be decomposed into two peaks at about 289.6 and 284.5 eV, which bear relationship to carbon and residual carbon containing lithium impurities such as Li_2_CO_3_. These results are consistent with those of the literature. Li_2_CO_3_ film is known to form on the surface of Ni-rich layered oxides due to reaction with atmospheric CO_2_ [40,41]. On the basis of these XPS analyses, we conclude that the surface layer present on NCM811 is a few nanometers thick and that it is mainly constituted of Li_2_CO_3_.

### 3.3. Raman Scattering Spectroscopy

**Figure 3:**
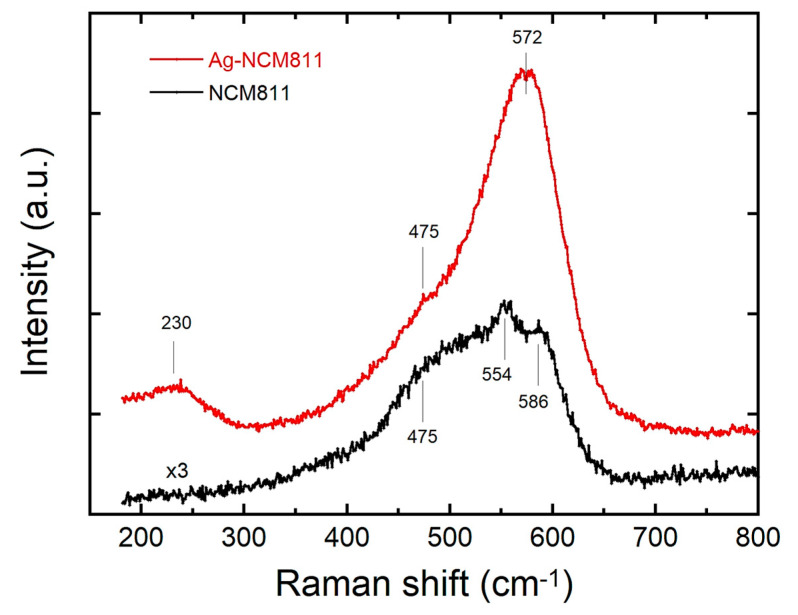
Raman spectra of pristine NCM811 (black line) and Ag-NCM811 (red line). NCM811 spectrum intensity is magnified by ×3 for clarity. Raman spectroscopy measurements were performed using a Renishaw inVia confocal Raman microscope system with a 532 nm excitation laser.

Raman scattering (RS) spectroscopy was used to ascertain the local structure, surface state, and composition of the as prepared samples [42]. It is mainly a surface probe analysis that is sensitive to the short-range environment of oxygen coordination around the cations in oxide frameworks. As shown in Figure 3, the Raman spectrum of pristine NCM811 displays three main bands located at 475, 554 and 586 cm^−1^, which corresponds to the active modes of the layered Li*M*O_2_ (*M* = Ni, Mn, or Co) lattice (*D*_3*d*_^5^ spectroscopic symmetry). This vibrational pattern confirms the good crystallinity and the rhombohedral crystal structure of LiNi_0.8_Co_0.1_Mn_0.1_O_2_ and supports the XRD results. The irreducible representation from group theory was used to predict the Raman-active vibrational modes of layered Li*M*O_2_ (*M* = Ni, Mn, or Co) compounds, in which the representation of the vibration modes associated to each transition-metal ion are as follows Г = 2*A*_2u_ + 2*E*_2u_ + *A*_1g_ + *E*_g_. Only the *A*_1g_ and *E*_g_ are Raman active modes and originate from the *M*–O stretching and O–*M*–O bending modes, respectively. As LiNi_0.8_Co_0.1_Mn_0.1_O_2_ has three different transition-metal cations (*M* = Ni + Mn + Co) located in 3*b* Wyckoff site of the rhombohedral structure, the spectral deconvolution using bands can be performed by three pairs (*A*_1g_ + *E*_g_) of Raman modes. The best fit to the Raman spectrum was achieved by starting from a prescribed set of three individual sets of Lorentzian shape (Figure S5). These vibrational features overlap to produce the broad band located in the range of 400–650 cm^−1^, which is characteristic of the Raman spectrum of Ni-rich oxide materials [43,44]. Table S1 lists the results (positions, bandwidth and band area) of the deconvoluted spectra. These values match well with those of single-layered compounds LiNiO_2_, LiCoO_2_ and LiMnO_2_ [45].

The Raman spectrum of Ag-NCM811 exhibits a different shape compared to that of pristine material. Three main bands are observed at 230, 475 and 572 cm^−1^, which indicates a significant structural change in the vibrational modes at the surface of Ag-coated crystals. A careful analysis of the Ag-NCM811 Raman spectrum shows that it can be decomposed in three components: (i) the fundamental modes of NCM811 lattice (*D*_3*d*_^5^ symmetry) with the characteristic peak at 475 cm^−1^ (*E*_g_ mode of O-Ni-O bonds), (ii) the broad band at 230 cm^−1^ assigned to the lattice mode of the monoclinic AgO phase, and its broadening (~50 cm^−1^) possibly due to the amorphous AgO covering the sample, and (iii) the strong band at 572 cm^−1^ corresponding to the presence of Ag-O on the particle surface, where a single Raman active vibration of *T*_2g_ symmetry at 565 cm^−1^ is the only predictive vibration expected of silver (I) oxide based on its symmetry considerations [46]. On the other hand, a similar spectral deconvolution using Lorentzian bands shows that the bandwidths of the *ν*_1_ and *ν*_2_ of Ni-type vibrations for the NCM811 sample are slightly larger than those of Ag-NCM811. A possible explanation for the broadening of the Raman bands for Ni-containing compounds is the Ni_Li_ antisite defect (Ni on Li site), which supports the results of XRD of larger Ni/Li cationic disorder in the pristine sample. The enhanced Raman intensity of Ag-NCM811 is attributed to the presence silver at the particle surface inducing a surface-enhanced Raman scattering (SERS). It is well known that silver and gold nanoparticles enhance the Raman signal of molecules near them, although they are not Raman-active. In other words, what we observe is not the Raman signal of silver–silver bonds but rather the enhancement of signals from molecules in close proximity, sometimes by many orders of magnitude [47].

### 3.4. Morphology

**Figure 4:**
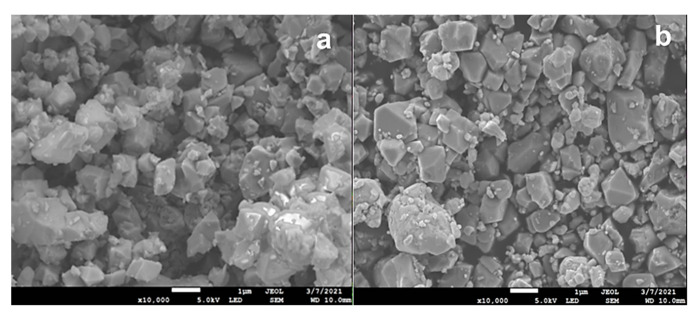
SEM image of (**a**) pristine NCM811 and (**b**) Ag-NCM811 samples (scale bar = 1 µm).

**Figure 5:**
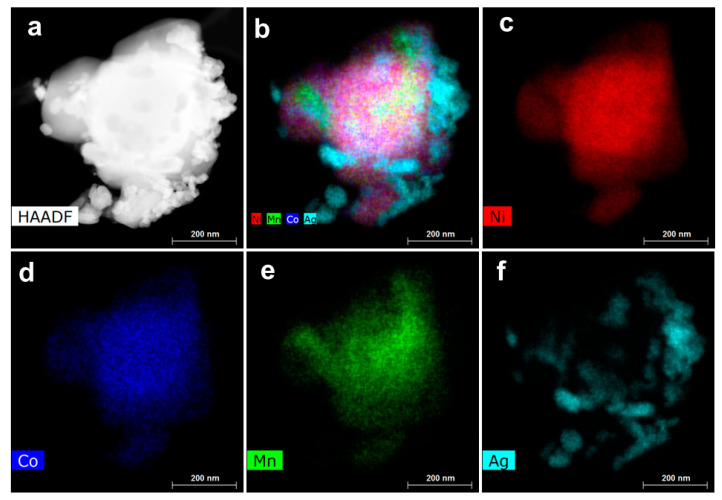
(**a**) HAADF-STEM image and (**b**–**f**) EDS elemental mapping images. (**b**) Overlay, (**c**) Ni, (**d**) Co, (**e**) Mn, and (**f**) Ag illustrating the core–shell structure of the of Ag-NCM811 sample.

The morphology of the pristine and surface-modified samples was implemented using SEM, HRTEM, and HAADF-STEM measurements. Figure 4 displays SEM images of both samples, showing faceted particles with a size distribution ranging from 0.5 µm to 2.0 µm. No significant difference was observed in the structural appearance, suggesting that a small amount of coating does not affect the morphology of these materials. The HRTEM images (Figure S5) visualize the atomic lattice in the crystalline NCM811 material. The images show consistent contrast throughout the whole observation area, revealing a single solid structure without any crystal dislocations. Perfect lattice fringes with a *d*-space of 0.47 nm correspond to the (003) planes in the layered lattice of the NCM811 oxide. The HRTEM image of Ag-NCM811 reveals that the Ag deposit is 1–2 nm thick. Figure 5 shows the high-angle annular dark-field scanning transmission electron microscopy (HAADF-STEM) image and the EDS elemental mapping images of Ni, Co, Mn, and Ag illustrating the core–shell structure of the of Ag-NCM811 sample. Compared to the bare sample, a clear deposited layer is observed on the surface of the coated sample. The EDS elemental mapping suggests that Ni, Mn and Co are distributed homogeneously on the NCM811.

### 3.5. Microporosity

**Figure 6:**
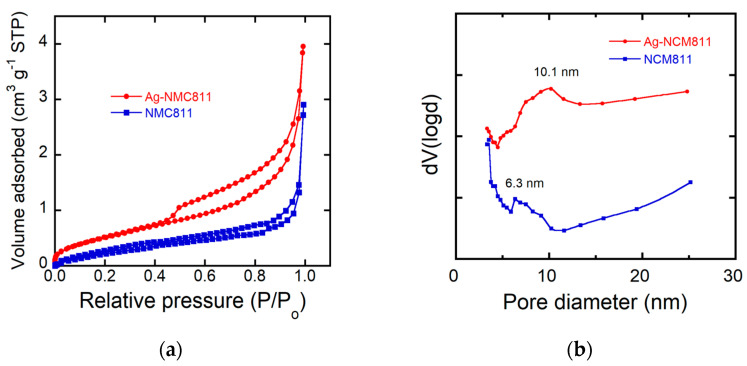
(**a**) N_2_ adsorption–desorption of pristine NCM811 and Ag-coated NCM811 samples. (**b**) Pore size distribution.

Microporosity of NCM811 samples was implemented via BET analysis of the adsorption/desorption isotherms (Figure 6a), providing specific surface area (*S_BET_*) and pore size. The pore size distribution of samples is displayed in Figure 6b. SSA value of pristine NCM811 is 1.13 m^2^ g^−1^ (pore size of 6.6 nm) compared to 2.03 m^2^ g^−1^ (pore size of 10.1 nm) for Ag-NCM811, which reflects a significant increase in surface roughness of NCM particles upon Ag coating. The equivalent particle size of the samples was calculated from the BET data and compared with the average size determined from SEM images. The average particle diameter (nm) is expressed by the relation [48]

(3) $$L_{BET}=\frac{6000}{d\times S_{BET}},$$

where *d* the gravimetric density (4.8 g cm^−3^ for LiNi_0.8_Co_0.1_Mn_0.1_O_2_). Results are listed in Table S2. The particle size values *L_BET_* and *L_SEM_* evaluated from BET and measured from SEM images, respectively, showed relatively good agreement.

The increase in SSA value for Ag-coated cathode material observed by BET measurements is attributed to the non-uniform coating including both metallic silver and “amorphous” silver oxide, which induce an increase in surface roughness and a significant increase in pore size (from 6.6 to 10.1 nm). It is well known that the porosity favors the electrochemical performance of the electrode as the interfacial surface between electrode and electrolyte enhances the Li-ion transport. This is confirmed by the larger charge transfer resistance measured via EIS experiments.

### 3.6. Electrochemical Properties

**Figure 7:**
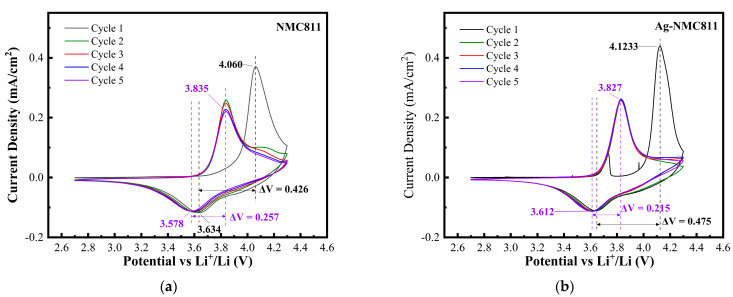
Cyclic voltammograms of pristine NCM811 (**a**) and Ag-NCM811 (**b**) electrodes recorded in the potential range of 2.7–4.3V vs. Li^+^/Li at low scan rate of 0.02 mV s^−1^.

The NCM811 and Ag-NCM811 cathode materials were electrochemically investigated using cyclic voltammetry (CV) and galvanostatic charge–discharge (GCD) techniques. Figure 7 displays the first five consecutive CV cycles recorded in the potential range of 2.7–4.3 V vs. Li^+^/Li at very slow scan rate of 0.02 mV s^−1^. As shown in Figure 7, both samples have the same characteristic redox peaks with a relatively high potential oxidation peak (>4.0 V) in the first cycle. This irreversible oxidation peak refers to an irreversible electrochemical reaction that usually occurring in the first cycle in Ni-rich materials. This behavior may be due to the Li_2_CO_3_ surface layer, which has poor ionic conductivity (~10^−9^ S cm^−1^) and no electronic conductivity, and may impede the electrochemical reaction, leading to a large increase in the overpotential. Li_2_CO_3_ film is known to form on the surface of Ni-rich layered oxides due to reaction with atmospheric CO_2_ [40]. Base on XPS analyses, we conclude that the surface layer present on NCM811 is a few nanometers thick and that it is mainly constituted of Li_2_CO_3_. As previously demonstrated by Aurbach et al. [41] and more recently by Grenier et al. [49], the relatively high potential oxidation peak (>4.0 V) observed in the first cycle can be attributed to the Li_2_CO_3_ surface layer. An interesting phenomenon occurs during the oxidation of the first cycle in the Ag-NCM811 sample. The CV curve seems to overlap with the next four cycles until the potential reaches 3.7 V, after which the current suddenly drops to almost zero. We believe that the shift from 3.6 to 3.7 V occurs mainly due to the lithium intercalation reaction through the surface spots that are not covered by Li_2_CO_3_. These spots without Li_2_CO_3_ could be due to the lack of silver on some parts of the surface. For the initial stage, the surface areas covered by Ag cannot react with atmospheric CO_2_ in air. At about 3.7 V, the products of oxidation reaction of Li_2_CO_3_ could cover those spots and impede the deintercalation reaction. After this early step, the reaction becomes similar to that shown in the first oxidation of uncoated NCM811. It was shown that upon cycling, the surface chemistry of these electrodes changes considerably. It seems that the first cycling helps to remove the pristine Li_2_CO_3_ film, thus enabling further massive reaction of the active mass with solution species. Note that after the first oxidation, both NCM811 and Ag-NCM811 electrodes do not show significant increase in polarization during reduction and following cycles. This is because the Li_2_CO_3_ layer dissolves or breaks down to create fast conduction pathways. As shown in Figure 7b, no significant change or additional redox peak are detected after the first cycle, indicating that the Ag coating has no effect on the redox reaction occurring in the layered structure of LiNi_0.8_Co_0.1_Mn_0.1_ O_2_. The anodic and cathodic peaks of NCM811 and Ag-NCM811 located at 3.82/3.60 V and 3.81/3.62 V, respectively, are attributed to the redox reactions between Ni^2+^, Ni^3+^ and Ni^4+^ during the charge–discharge process [50,51]. The potential interval (Δ*E*_OR_) is an indicator for the electrode reaction reversibility (low value) and for the degree of the electrochemical polarization (high value) [52]. The potential difference Δ*E*_OR_ *=* 0.19 V for Ag-NCM811 is smaller than that of NCM811 with Δ*E*_OR_ = 0.22 V. This shows the positive effect of the Ag coating layer on the kinetics of the cathode material [53], providing better Li^+^ conduction and inhibiting electrode polarization during the charge–discharge cycling process [54]. Furthermore, for five cycles, the redox peaks of the Ag-NCM811 electrode are sharper and better overlapped than those of the NCM811 electrode, which confirms the positive effect of Ag coating.

**Figure 8:**
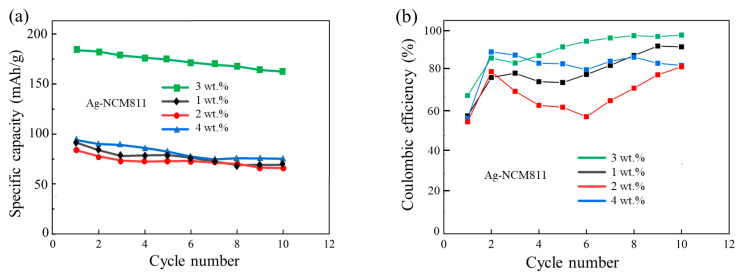
Comparison of the electrochemical performance of Ag-NCM811 electrodes containing various amounts of Ag (1, 2, 3, and 4 wt.%). (**a**) Specific discharge capacity upon cycling. (**b**) Coulombic efficiency vs. cycle number.

A systematic study was carried out on 1, 2, 3 and 4 wt.% Ag-coated NCM811 electrodes to determine the effect of the load capacity of Ag nanoparticles on the performance of the material. Voltage–capacity and Coulombic efficiency plots are presented in Figure 8 for the ten first cycles at 0.1 C. Results show clearly that the 3 wt.% Ag-coated NCM811 sample prepared with 3 mol.% ethanolic silver nitrate solution exhibits the best electrochemical performance.

**Figure 9:**
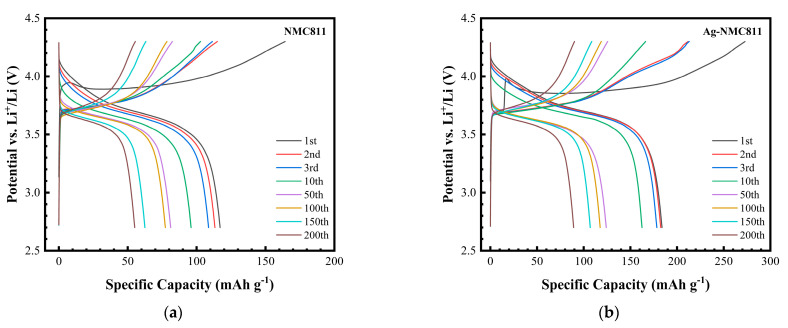
Galvanostatic charge–discharge profiles of (**a**) pristine NCM811 and (**b**) Ag-NCM811 recorded at 0.1 C within the voltage range 2.7–4.3 V.

Figure 9 shows a series of galvanostatic charge–discharge cycles carried out at a 0.1 C rate for pure NCM811 and coated 3 wt.% Ag-NCM811 electrodes in the potential range of 2.7–4.3 V vs. Li^+^/Li. Both electrodes present similar smooth curves with charge and discharge plateaus at about 3.85 and 3.70 V, respectively. The first charge curve of Ag-NCM811 shows a plateau at 3.7 V and the curve suddenly increases to about 3.9 V at about 20 mAh g^−1^ capacity. The plateau during the first charge is in accordance with the CV result shown in Figure 7b, and it is attributed to the detachment of Ag nanoparticles and the oxidation of Li_2_CO_3_ during the first charging, as mentioned before. Figure S7 presents the voltage–capacity profiles of cathodes containing various amounts of silver, i.e., 1, 2 and 4 wt.%. The capacity at 3.7-V plateau is highly correlated to the Ag loading. These results show that the reaction at this plateau should have Ag involved and that the 3.7-V plateau belongs to the first charging Ni^2+/3+, 4+^ reactions. It is possible that the Ag coating allowed the Ni reactions without undergoing the Li_2_CO_3_ coating and the oxidation of Li_2_CO_3_. These results consistently show a repeatable potential plateau at around 3.7 V in agreement with the peak observed in cyclic voltammograms. As previously demonstrated in Ref. [49], an electronic or ionic barrier would increase the impedance of the cathode. In the early stage of the first charge reaction, the resistive Li_2_CO_3_ layer leads to a high overvoltage. After the first charging, there is no difference regarding plateaus for both coated and uncoated samples, which indicates that no extra electrochemical reaction occurred due to the action of the coating layer. The similarity provides evidence that coating layer does not block Li^+^ sites during insertion/extraction process and no phase transition occurs (from layered *R*$\bar{3}$*m* to spinel *Fd*$\bar{3}$*m* and even to rock-salt phase *Fm*3*m*) associated with the production of side reactions between the NMC electrode and electrolyte and lowering the average potential [55].

**Figure 10:**
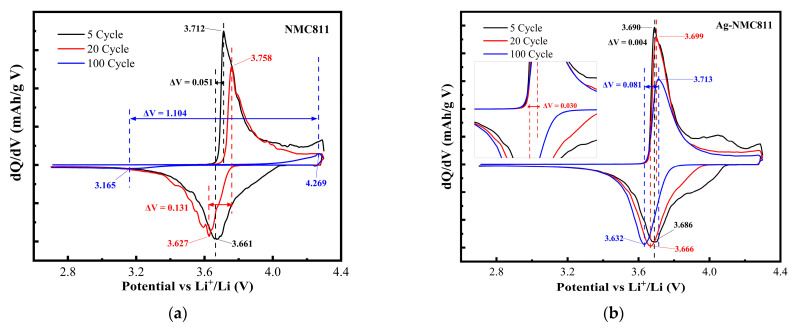
d*Q*/d*V* vs. *V* plots for pristine NCM811 (**a**) and Ag-NCM811 (**b**) electrodes. Cycles 5, 20, and 100 are shown.

Electrochemistry is inherently more sensitive than structural characterization methods such as XRD for detecting changes in electroactive phases. Thus, differential capacity (d*Q*/d*V*) vs. *V* plots are important indicators for tracking the phase retention during cycling. It is worth noting that in such a plot, peaks correspond to plateaus in the GCD profiles. Figure 10 depicts the differential capacity for both electrodes. Regarding the case of pristine NCM811, all peaks decrease throughout cycling, indicating the electrochemical fading of this electrode. The peak potential difference is Δ*V* = 1.10 V after 100 cycles (Figure 10a). In contrast, the Ag coating of NCM811 results in retention of the differential capacity peaks with Δ*V* = 0.03 V after 100 cycles, mitigating the fading mechanisms in this electrode (Figure 10b).

**Figure 11:**
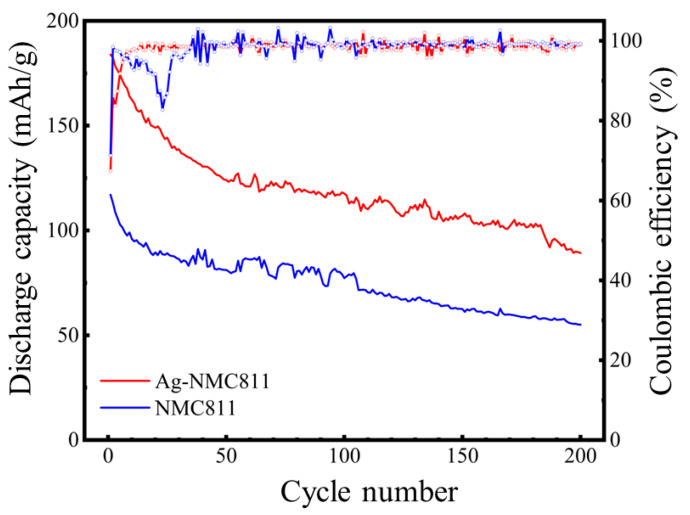
Specific discharge capacity as a function of cycle number of bare NCM811 and Ag-NCM811 at a current density of 0.1 C in the potential range 2.7–4.3 V.

Figure 11 presents the cycling properties of NCM811 and Ag-NCM811, tested at 0.1 C rate in the voltage range 2.7–4.3 V. Both samples exhibit a decrease in discharge capacity upon cycling. The results show that coating layer stabilizes the structure through minimizing the change in the valence states of nickel and manganese during Li^+^ intercalation/deintercalation. Additionally, the results can also be used to determine the stability of the layered structure and the depth of lithium removal leading to voltage decay in the cycling process [56]. The improvement of the stability associated to the Ag coating is confirmed by the slower voltage decay and polarization observed for Ag-NCM811 compared to that of pristine NCM811. It is noteworthy that the charge–discharge results match well with CV results mentioned previously. Both samples have nearly the same initial charge capacity of 275 mAh g^−1^ with different discharge capacity values. Ag-NCM811 sample presents a higher initial discharge capacity of 184 mAh·g^−1^ at the first cycle with a Coulombic efficiency of 67.41% and discharge capacity can reach 183 mAh·g^−1^ at the second cycle, corresponding to a lower fading rate of 0.76%. In contrast, the pristine NCM811 delivers a lower initial discharge capacity of 117 mAh·g^−1^ at the first cycle with a Coulombic efficiency of 71.28% and a discharge capacity of 113 mAh·g^−1^ at the second cycle, corresponding to a higher fading rate of 3.16%. With subsequent cycling, the Ag-NCM811 delivers discharge capacities of 120 mAh·g^−1^ at the 100th cycle and 90 mAh·g^−1^ at the 200th cycle, respectively. In addition, the discharge capacity of pristine NCM811 reaches 77 mAh·g^−1^ at the 100th cycle and 55 mAh·g^−1^ at the 200th cycle, respectively. This enhancement in electrochemical properties of Ag-NCM811 sample may be attributed to silver coating that prevents violent oxidation and decomposition of electrolyte at high voltage. The Ag coating also blocks the direct contact and side reaction between the generated HF in electrolyte and the material surface, which alleviates the dissolution of the bulk material and protects the surface of particles from the corrosion reactions. Moreover, it provides a better path for charge transfer and Li transport, thus accelerating the rate of lithium-ion diffusion and improving the material dynamic process and decreasing the polarization gap of electrodes. Additionally, the coating layer absorbs stress to reduce the volume change and structural change, including irreversible phase transformation from layered to spinel or even salt-rock phase, in the process of charge and discharge [57,58]. Table S3 presents a comparison of the electrochemical properties of Ag-coated NCM811 with previously coated NCM811 cathode materials as a function of the operating potential window [59,60,61].

### 3.7. EIS Measurements

Electrochemical impedance spectroscopy is a useful in situ method to examine changes in the transport parameters of electrodes, including electrode–electrolyte interface resistance and Li^+^ ion kinetics. To further shed light on the electrochemical properties of NCM811 and Ag-NCM811 electrode materials, EIS measurements were carried out before cycling with fresh cells and after 200 cycles recorded at a 0.1 C rate; Nyquist plots are shown the in Figure S8a,b, respectively. The equivalent circuit model used to analyze the EIS patterns is shown in Figure S8c, which is composed of four components: the ohmic resistance of the cell (*R*_s_), the impedance of the SEI layer (parallel *R*_SEI_-*CPE*_SEI_ circuit), the charge transfer impedance and interfacial capacitance at the electrode–electrolyte interface (parallel *R*_ct_-*CPE*_dl_ circuit), and the Warburg impedance (*Z*_w_), which characterizes the Li^+^ ion diffusion-controlled process [62]. The intercept at the real part (*Z*′) axis at highest frequency stands for the solution resistance (*R*_s_). The spectra in the mid-frequency region are usually semicircular in shape and related to the charge transfer resistance (*R*_ct_) caused by the Faradaic reactions and the double-layer capacitance (*C*_dl_) on the grain surface (parallel *R*_ct_-*C*_dl_ circuit). *C*_dl_ is represented by a constant phase element (*CPE*), which models the behavior of an imperfect capacitor or the non-uniform charge distribution at the grain interfaces and describes the deformed nature of the semicircle in Nyquist plots. The values of the elements from the equivalent circuit model were obtained by the following formulas used for simulating the EIS for the charge-transfer reaction [63]:

*Z_R_*_ct_ = *R*_ct_, (4)

(5) $$Z_{CPE}\left(\omega \right)=\frac{1}{T}\;{\left(j\omega \right)}^{-p},$$

where *T* is the *CPE* coefficient, *p* the *CPE* exponent, *j* the unit of the complex number (√ − 1) and *ω* denotes the angular frequency. A *CPE* represents a resistor when *p* = 0, a capacitor with capacitance of *C* when *p* = 1, an inductor when *p* = −1, and a Warburg impedance when *p* = 0.5 [64]. In the low-frequency region, the linear part is attributed to the frequency dependence of ion transport/diffusion in the host. Therefore, the “infinite” Warburg impedance is expressed as [65]

(6) $$Z_{w}=Z_{w-R}\omega^{-\frac{1}{2}}\left(1-j\right).$$

From fitted parameters listed in Table 2, the general trend is an increase in the total impedance after 200 cycles at 0.1 C rate for both electrodes.

The relatively high value of the initial internal resistance (*R*_s_) is attributed to the freshly assembled cell with uncycled electrodes. *R*_SEI_ and *R*_ct_ significantly increased during cycling, as shown in GCD curves with an increase in the cell polarization after long-life cycling. The real part *Z*′(ω) of the total impedance of the cell is the sum of the real part of the four components:

*Z*′(ω) = *R*_s_ + *R*_SEI_ + *R*_ct_ +*Z*_w−R_. (7)

**Figure 12:**
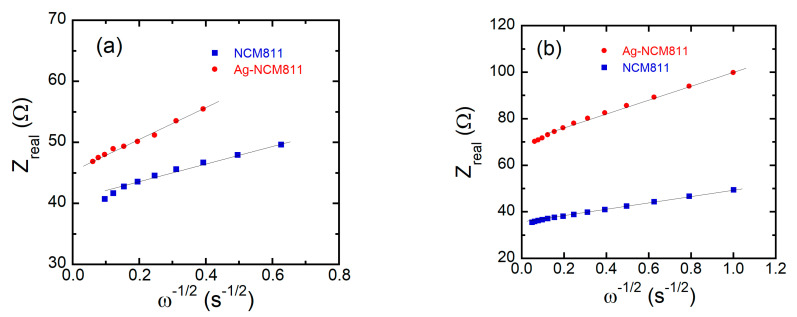
Plots of the real part of the impedance vs. ω^−1/2^ for bare NCM811 and Ag-NCM811 electrodes before (**a**) and after 200 cycles (**b**) at 0.1 C rate. Full lines are the slope of the *Z*_real_ vs. ω^−1/2^ fits providing the Warburg impedance.

*Z*_w−R_ can be determined from the slope of the Warburg plot *Z*′ vs. ω^−1/2^ (Figure 12) and the apparent diffusion coefficient *D*_Li_ is obtainedp0 according to the relation [66]

(8) $$D_{\mathrm{Li}}=\frac{R^{2}T^{2}}{2A^{2}n^{4}F^{4}C_{\mathrm{Li}}{}^{2}Z_{w-R}{}^{2}},$$

in which *R* is the gas constant, *T* is the absolute temperature, *F* is the Faraday’s constant, *n* is the number of electrons transferred, *C*_Li_ is the concentration of Li^+^-ion inside the electrode, and *A* is the effective surface area of the electrode. *D*_Li_ of 6.2 × 10^−12^ cm^2^ s^−1^ for the fresh Ag-NCM811 electrode is slightly higher than that of the bare NCM811 electrode (4.0 × 10^−12^ cm^2^ s^−1^). However, after 200 cycles, *D*_Li_ of Ag-NCM811 decreases to 5.4 × 10^−13^ cm^2^ s^−1^. This comparison confirms that the Ag coating facilitates the Li^+^ ion kinetics due to several factors such as better charge-transfer resistance (19.8 vs. 25.2 Ω) for Ag-NCM8111, the lower Li/Ni cation mixing, and the increased structural stability. After long-life cycling (200 cycles at a 1 C rate), the diffusion coefficients of Li^+^ ion for both electrodes decrease from ~10^−12^ to ~10^−13^ cm^2^ s^−1^ due to electrode aging.

## 4. Discussion

The improvement in calendar and cycling life of a Li-ion cell is a challenge that requires sophisticated technology to prevent degradation of electrochemical reactions. A part of the solution is the surface modification of the electrode materials. It is widely accepted that a coating layer can improve the structural stability and mitigate side reactions. In addition, in many cases, the coating layer can decrease the disorder of cations in crystal sites and enhances the ion/electron transport. These beneficial coating effects can also be expected with the use of a very small quantity of noble metals, such as silver, which can be deposited very easily using a low-cost nitrate silver solution.

As far as we know, the literature reports only one work related to the Ag coating NCM811 by Sun et al. [27]. It was claimed that an amorphous Ag coating layer is formed, but there is no accurate demonstration of the morphology nor composition of this surface layer. In our case, we demonstrate clearly that both metallic Ag and silver oxide (AgO) layers are deposited on the surface of NCM811 nanoparticles as a non-uniform coating. Additionally, the green synthesis used, i.e., sol–gel method assisted by EDTA chelating agent, provides well-crystallized faceted submicronic particles, while the co-precipitation used in Ref. [27] provides agglomerates.

This study introduces five novel aspects. (1) It explores the impact of silver coating on the structural integrity of the NCM811 layered lattice. (2) The sample shows well-crystallized particles with lower strain compared with the bare sample, archived with a small amount of ~3 wt.% Ag. (3) A combination of analytical techniques demonstrates that the Ag deposit is composed of Ag^0^ and Ag^+^ (oxide) phases with equal content covering homogeneously the active NCM811 particles. (4) The electrochemical properties of the Ni-rich cathode material prepared with the facile sol–gel synthesis reveal the minor degradation of the Ag-coated electrode compared with the bare NCM811. (5) This study quantifies the beneficial contribution of the Ag coating by revealing improved kinetics at long-cycling life (200 cycles), showing a decrease in the charge transfer resistance and an increase in the Li^+^ ion diffusion coefficient.

A comparison of the electrochemical performance of surface modified LiNi_0.8_Co_0.1_Mn_0.2_O_2_ composite electrodes reported in the literature is presented in Table 3. Only one group of researchers reports the Ag coating of NCM811 [27,28]. Their GCD tests indicate that 1 wt.% Ag-coated NCM811 exhibits an initial discharge capacity of 209 mAh g^−1^ and superior capacity retention of 90.5% after 90 cycles between 2.8 V and 4.3 V, but the composition of the Ag coating is not well established. Nevertheless, the value of *D*_Li_ of 1 wt.% Ag-coated NCM811 has increased from 3.79 × 10^−12^ (3rd cycle) to 9.02 × 10^−12^ cm^2^ s^−1^ (100th cycle).

It is noteworthy that this study demonstrates the beneficial effect of surface coating despite the use of the common electrolyte 1.2 mol L^−1^ LiPF_6_ in EC:EMC (3:7 by weight) for economic reasons. However, a more sophisticated suitable electrolyte, such as 0.6 mol L^−1^ LiTFSI + 0.4 mol L^−1^ lithium bis(oxalato)borate + 0.05 mol L^−1^ LiPF_6_ dissolved in ethylene carbonate and ethyl methyl carbonate (4:6 by weight), was utilized in a different study, which showed excellent stability at a high charge cutoff voltage of 4.5 V [77]. In a cell with lithium metal anode, a discharge capacity larger than 220 mAh g^−1^ (846 Wh kg^−1^) and capacity retention higher than 80% after 1000 cycles at 2 C rate were achieved. To further improve the performance, adding B_2_O_3_ and producing a B_2_O_3_/Ag coating is suggested, as the B^3+^-doping surface triggers a reduction in a small amount of Ni^3+^ to Ni^2+^, and it inhibits the irreversible phase transitions and extension of microcracks in the NCM material [76,78]. Moreover, a synergic effect can be obtained by doping with 1% of Nb in addition to the coating. Simple Nb doping without coating provides a retention of 94.55% after 100 cycles at 1 C, and a good rate capability with a capacity of 151 mAh g^−1^ at 5 C [79].

## 5. Conclusions

In this study, we successfully applied an Ag coating to improve the electrochemical performance of NCM811 cathodes. The NCM811 materials were synthesized via the sol–gel method by using EDTA as a chelating agent. The Ag-coated NCM811 material was synthesized using 3% of silver by a facile, cost-effective, scalable, and convenient method. Analytical methods of characterization, including XRD Rietveld refinements, Raman spectroscopy, HAATF-STEM and XPS analysis, demonstrate that the surface coating is composed of metal Ag^0^ and silver(I) oxide, with a total content of ~3 mol.%. XRD and HRTEM results show that Ag remains at the surface of NCM811 particles and does not dope into the material, preserving the layered structure integrity. From XRD and Raman analyses, it is evident that Ag-NCM811 has less cation mixing than the pristine NCM811. XPS analysis shows that Ni 2p_3/2_ peak of Ag-NCM811 shifted towards higher binding energy (855.55 eV) than that of NCM811 (855.16 eV), which can be attributed to decreased Ni^2+^/Ni^3+^ ratio. This proves that more Ni^3+^ ions appear on the Ag-NCM811 electrode surface than on that of pristine NCM811. The electrochemical characterization shows the positive effect of conductive Ag coating layer on the kinetics of Ag-NCM811, providing better Li^+^ conduction and inhibiting electrode polarization during the charge–discharge cycling process. Ag-NCM811 delivers discharge capacities of 185 mAh·g^−1^ at the first cycle, 120 mAh·g^−1^ at the 100th cycle and 90 mAh·g^−1^ at the 200th cycle with capacity retention of 64.86% and 48.65%, respectively.

## Figures and Tables

**Table 1:** Results of Rietveld refinements for pristine NCM811 and Ag-NCM811.

Sample	NCM811	Ag-NCM811
Lattice parameters		
*a* (Å)	2.875 (3)	2.878 (1)
*C* (Å)	14.221 (7)	14.230 (2)
*c*/*a*	4.94 (5)	4.943 (2)
*V* (Å^3^)	102.13	102.09
*I_(_*_003)_*/I*_(104)_ *	1.27 ± 0.03	1.31 ± 0.03
*L*_c_ (nm)	55 ± 2	50 ± 2
ε × 10^−3^ (rd)	0.86	0.65
Reliability factors		
*R_p_* (%)	9.18	10.12
*R_wp_* (%)	11.86	13.69
*χ* ^2^	1.24	1.45
Ni^2+^ % in Li layer	5.78	3.61
Material fraction (mol%)		
NCM811	100	96.7
Ag	0	1.7
AgO	0	1.6

* peak intensity ratios were obtained from normalized patterns.

**Table 2:** Fitting results of Nyquist plots of NCM811 and Ag-NCM811 electrodes before and after 200 cycles at 0.1 C rate.

Parameters	NCM811		Ag-NCM811	
	Fresh Cell	After Cycling	Fresh Cell	After Cycling
*R*_s_ (Ω)	19.31	10.9	28.1	47.7
*R*_SEI_ (Ω)	-	23.5	-	47.0
*CPE*_SEI-T_	-	1.04 × 10^−5^	-	1.809 × 10^−2^
*CPE*_SEI-P_	-	0.85	-	0.005
*R*_ct_ (Ω)	13.5	25.2	17.4	19.8
*CPE*_dl-T_	2.9 × 10^−5^	1.1 × 10^−4^	2.77 × 10^−5^	7.5 × 10^−5^
*CPE*_dl-P_	0.85	0.98	0.78	0.41
*Z*_w-R_	7.6	50.8	6.1	25.7
*Z*_w-T_	0.016	9.5	0.0124	6.197
*Z*_w-P_	0.49	0.50	0.45	0.31
*D*_Li_^+^ (cm^2^ s^−1^)	4.0 × 10^−12^	1.1 × 10^−13^	6.2 × 10^−12^	5.4 × 10^−13^

**Table 3:** Comparison of electrochemical performance of NCM811 electrode coated with various substances. Number of cycles is given in parentheses.

Coating Material	C-Rate	Specific Capacity (mAh g^−1^)	Ref.
g-C_4_N_3_ (~20 nm thick)	1 C (200)	130	[67]
Mg-Al-LDO (5 nm thick)	0.5 C (100)	125	[68]
SiO_2_ (0.25 wt.%; 1–3 nm thick)	0.5 (100)	160	[16]
TiO_2_ (6 nm thick)	0.1 (100)	180	[69]
MoO_3_ (3 wt.%; 5 nm thick)	1 C (100)	175	[70]
SiO_2_ (20–40 nm thick)	1 C (300)	160	[71]
Li_3_VO_4_ (3 wt.%; ~5 nm thick)	1 C (100)	140	[72]
Li_2_O·2B_2_O_3_ (50–200 nm thick)	0.5 C/1 C (100)	199	[19]
Al_2_O_3_ (2 wt.%; 2 nm thick)	1 C (100)	168	[73]
LaFeO_3_ (0.5 wt.%; ~60 nm)	0.2 C (80)	130	[74]
Li_3_PO_4_ (2 wt.%) + PPy (3 wt.%)	1 C (200)	157	[60]
PANI-PVP (5–7 nm thick)	1 C (100)	178	[75]
Amorphous Ag (1 wt.%)	1 C (90)	170	[27]
Li_2_O·2B_2_O_3_ (0.3 wt.%)	1 C (100)	145	[76]
Ag/AgO (3 mol.%, 1–2 nm thick)	0.1 (100)	120	this work

## Data Availability

Not applicable.

## Supplementary Materials

The following supporting information can be downloaded at: https://www.mdpi.com/article/10.3390/mi14050907/s1, Figure S1. Schemes of sample preparation (A) sol–gel synthesis of pristine NCM811 and (B) in situ coating process of Ag on NCM811 particles; Figure S2. (a–c) Enlarged XRD patterns of pristine NCM811 (red) and Ag-NCM811 (blue); Figure S3. X-ray diffractogram of NCM811 compared with that of cubic Ag (JCPDS card No. 89-3722). Figure S4. Typical spectral deconvolution of the Raman pattern of NCM811. Table S1. Analysis of the Raman active modes *E*_g_ and *A*_1g_ for NCM811 and Ag-NCM811 samples using Lorentzian profiles. Band positions are provided with an accuracy of ±1 cm^−1^. Figure S5. HRTEM images of pristine NCM811 (a) and Ag-NCM811 (b). Table S2. Brunauer–Emmett–Teller (BET) specific surface area (*S*_BET_), pore volume, pore size, and calculated average particle size from BET data (*L*_BET_) using Equation (3), mean particle size measured using SEM imaging (*L*_SEM_) for pristine and Ag-coated NCM811 samples. Table S3. Comparison of the electrochemical properties of Ag coated NCM811 with previously coated NCM811 cathode materials as a function of the operating potential window. Figure S6. EIS measurements. Nyquist plot of pristine NCM811 and Ag-NCM811 electrodes for (a) fresh cells and (b) after 200 cycles at 1 C rate. (c) Equivalent circuit model used for simulating the Nyquist plots. *R*_s_ and *R*_ct_ are the solution resistance and charge-transfer resistance, respectively. Parallel *R*_SEI_-*CPE*_SEI_ represents the SEI layer impedance and *CPE*_dl_ represents the double-layer capacitance. Z_W_ represents the Warburg impedance. Figure S7. Gavanostatic charge-discharge profiles recorded at 0.1C rate of Ag-NCM811 electrodes containing various amounts of Ag: (a) 1 wt.%, (b) 2 wt.% and (c) 4 wt.%. Table S4. Comparison of the electrochemical properties of Ag coated NCM811 with previously coated NCM811 cathode materials as a function of the operating potential window. Figure S8. EIS measurements. Nyquist plot of pristine NCM811 and Ag-NCM811 electrodes for (a) fresh cells and (b) after 200 cycles at 0.1C rate. (c) Equivalent circuit model used for simulating the Nyquist plots. Rs and Rct are the solution resistance and charge-transfer resistance, respectively. Parallel RSEI-CPESEI represents the SEI layer impedance and CPEdl represents the double-layer capacitance. ZW represents the Warburg impedance.
