# Supplementary material for: Silver Nanocoating of LiNi0.8Co0.1Mn0.1O2 Cathode Material for Lithium-Ion Batteries
Source: Micromachines (Basel). 2023 Apr 23;14(5):907. doi: 10.3390/mi14050907 (PMC10221140; doi:10.3390/mi14050907)
Supplement: Supplementary file 1 [file micromachines-14-00907-s001.zip › micromachines-2282416-supplementary.pdf]

Supplementary Information

Silver Nanocoating of $LiNi_{0.8}Co_{0.1}Mn_{0.1}O_2$ Cathode Material for Lithium-Ion Batteries

Xintong Li [1], Kai Chang [1], Somia Abbas [2], Rasha S. El-Tawil [2], Ashraf E. Abdel-Ghany [2], Ahmed M. Hashem [2,*], Hua Wang[1], Amanda Coughlin[3], Shixiong Zhang[3,4], Alain Mauger [5], Likun Zhu [1,*] and Christian M. Julien[5,*]

[1] Department of Mechanical and Energy Engineering, Indiana University-Purdue University Indianapolis, Indianapolis, IN 46202, USA

[2] National Research Centre, Inorganic Chemistry Department, Behoes Street, Dokki, Giza 12622, Egypt

[3] Department of Physics, Indiana University, Bloomington, IN 47405, USA

[4] Quantum Science and Engineering Center, Indiana University, Bloomington, IN 47405, USA

[5] Institut de Minéralogie, de Physique des Matériaux et Cosmologie (IMPMC), Sorbonne Université, UMR-CNRS 7590, 4 Place Jussieu, 75752 Paris, France

* Correspondence: ahmedh242@yahoo.com (A.M.H.); likzhu@iupui.edu (L.Z.); christian.julien@sorbonne-universite.fr (C.M.J.)

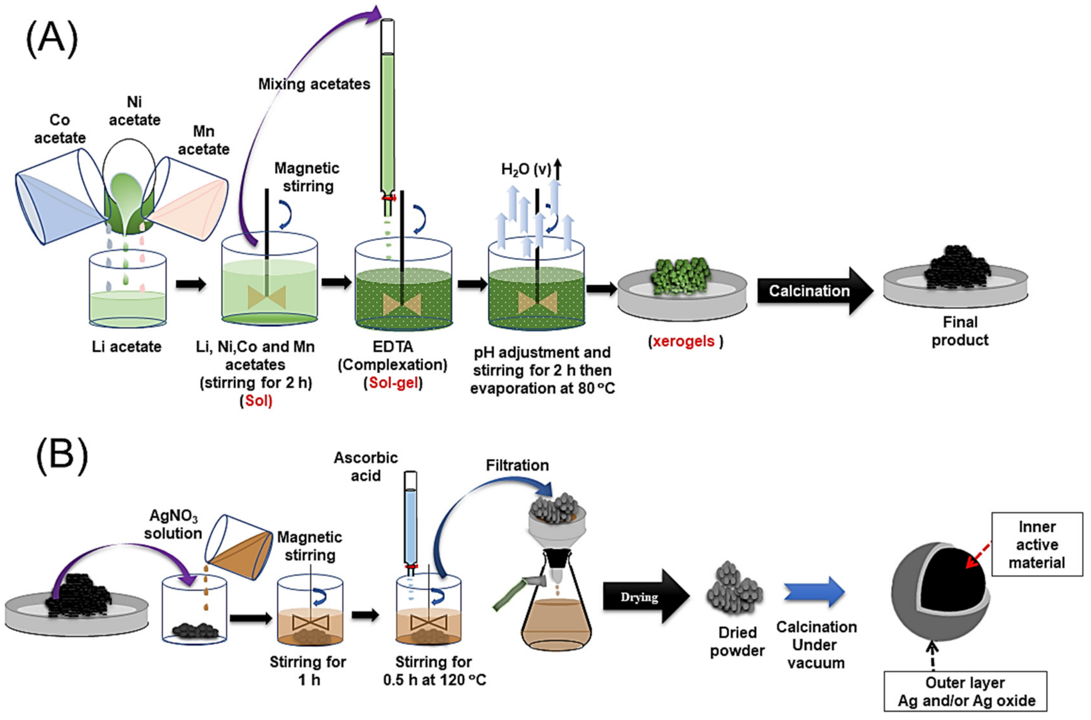

Figure S1. Schemes of sample preparation (**A**) sol-gel synthesis of pristine NCM811 and (**B**) in-situ coating process of Ag on NCM811 particles.

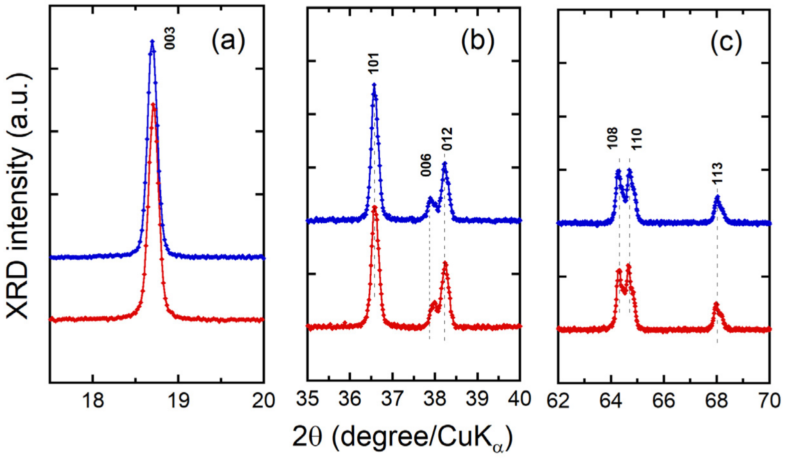

Figure S2. (**a-c**) Enlarged XRD patterns of pristine NCM811 (red) and Ag-NCM811 (blue).

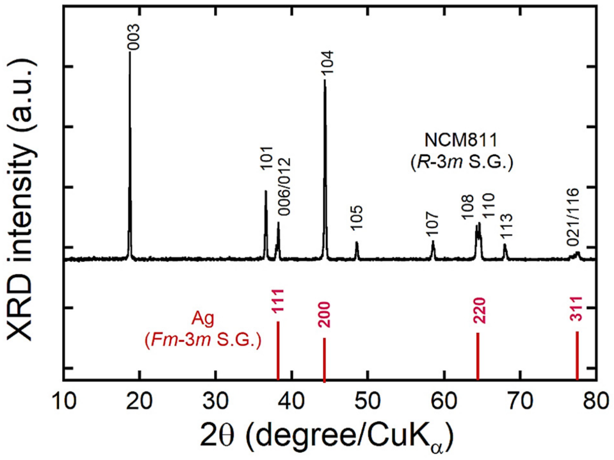

Figure S3. X-ray diffractogram of NCM811 compared with that of cubic Ag (JCPDS card No. 89-3722).

Table S1. Analysis of the XPS response of the O 1s core level for pristine and Ag-coated NCM811.

Material	Lattice oxygen (%)	Surface defect oxygen (%)
Pristine NCM811	86.75	13.25
Ag-NCM811	89.10	10.90

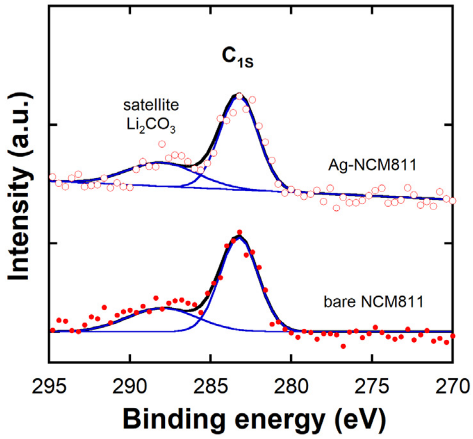

Figure S4. High-resolution XPS spectra of C 1s.

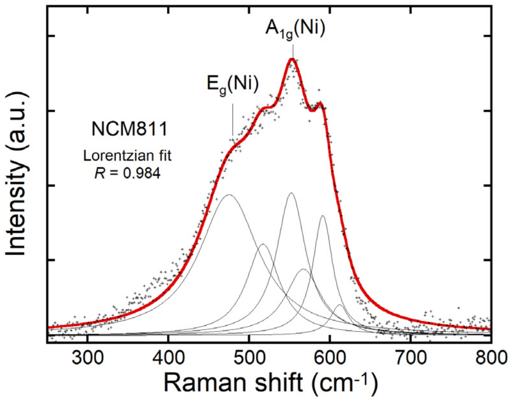

Figure S5. Typical spectral deconvolution of the Raman pattern of NCM811.

Table S2. Analysis of the Raman active modes E_g and A_{1g} for NCM811 and Ag-NCM811 samples using Lorentzian profiles. Band positions are given with an accuracy of ±1 cm^{-1}.

Modes		Band position (cm^{-1})	Band width (cm^{-1})	Band area
NCM811				
ν_1	E_g (Ni)	475	45	140459
ν_2	A_{1g} (Ni)	554	22	62507
ν_3	E_g (Co)	517	25	67614
ν_4	A_{1g} (Co)	567	27	2578
ν_5	E_g (Mn)	591	15	22934
ν_6	A_{1g} (Mn)	612	14	9199
Ag-NCM811				
ν_1	E_g (Ni)	475	38	430053
ν_2	A_{1g} Ni)	556	19	69299
ν_3	E_g (Co)	514	26	98480
ν_4	A_{1g} (Co)	565	23	33288
ν_5	E_g (Mn)	589	15	64825
ν_6	A_{1g} (Mn)	611	15	3961

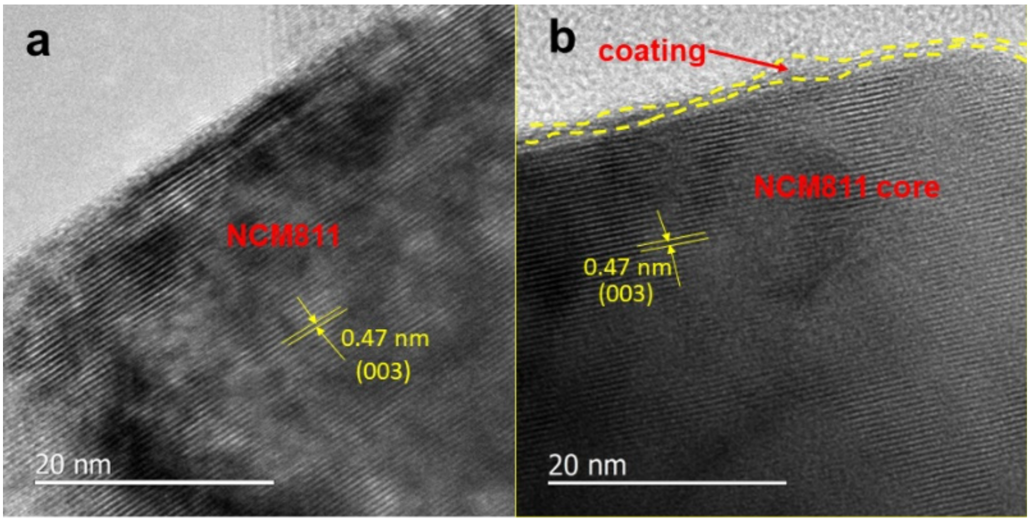

Figure S6. HRTEM images of pristine NCM811 (**a**) and Ag-NCM811 (**b**).

Table S3. Brunauer–Emmett–Teller (BET) specific surface area (S_{BET}), pore volume, pore size, and calculated average particle size from BET data (L_{BET}) using Eq. (3), mean particle size measured using SEM imaging (L_{SEM}) for pristine and Ag-coated NCM811 samples.

**Samples**	S_{BET} (m^2 g^{-1})	L_{BET} (μm)	**Pore size (nm)**	L_{SEM} (nm)
Pristine NCM811	1.13	1.1	6.3	1.2
Ag-NCM811	2.03	0.6	10.1	0.9

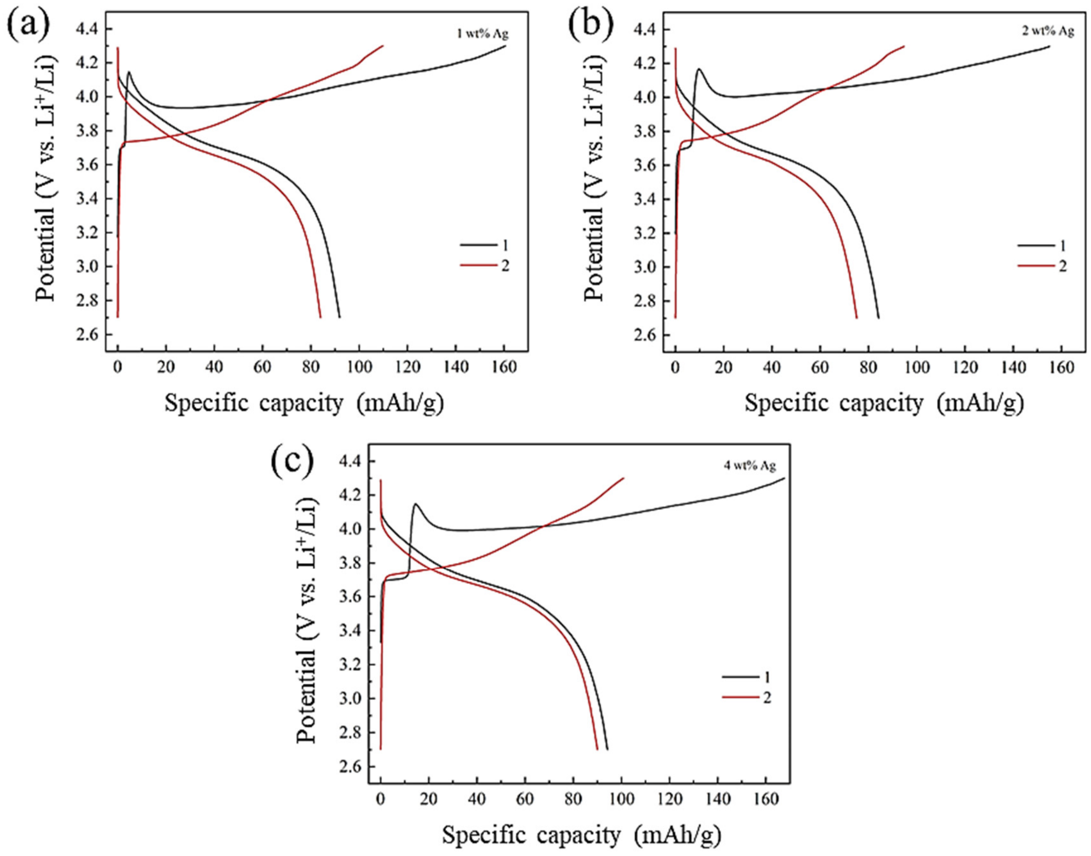

Figure S7. Gavanostatic charge-discharge profiles recorded at 0.1C rate of Ag-NCM811 electrodes containing various amounts of Ag: (**a**) 1 wt.%, (**b**) 2 wt.% and (**c**) 4 wt.%.

Table S4. Comparison of the electrochemical properties of Ag coated NCM811 with previously coated NCM811 cathode materials as a function of the operating potential window.

**Coatings**	**Potential window (V)**	**Initial discharge capacity ($mAh.g^{-1}$)**	**Capacity retention ratio (%) (rate, cycle number)**	**Ref.**
Li_3PO_4-$AlPO_4$-$Al(PO_3)_3$	3.0 - 4.3	201	85.4 (@0.1C, 50)	[1]
Li_3PO_4 and PPy	2.8 - 4.5	203	95.1 (@0.1C, 50)	[2]
CaF_2	2.7 - 4.3	149	79.68 (@0.1C,50)	[3]
Ag^0/Ag oxide	2.7 - 4.3	185	64.8 (@0.1C,100)	this work

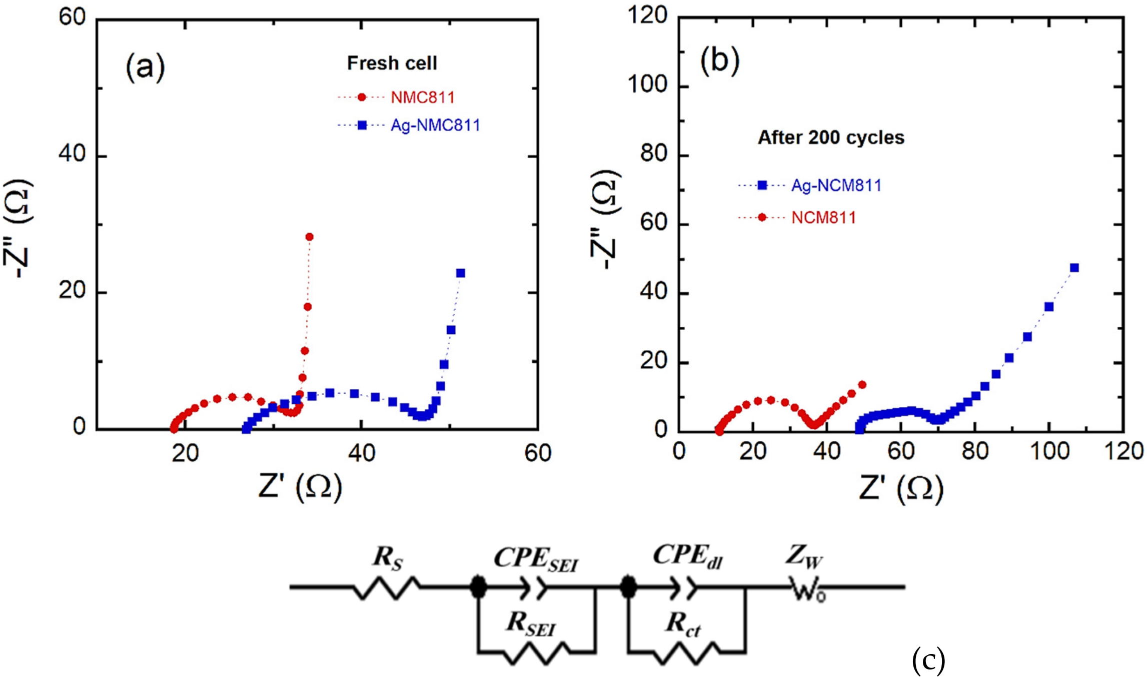

Figure S8. EIS measurements. Nyquist plot of pristine NCM811 and Ag-NCM811 electrodes for (**a**) fresh cells and (**b**) after 200 cycles at 0.1C rate. (**c**) Equivalent circuit model used for simulating the Nyquist plots. R_s and R_{ct} are the solution resistance and charge-transfer resistance, respectively. Parallel R_{SEI}-CPE_{SEI} represents the SEI layer impedance and CPE_{dl} represents the double-layer capacitance. Z_W represents the Warburg impedance.

References

1. Feng, Z.; Rajagopalan, R.; Sun, D.; Tang, Y.; Wang, H. In-situ formation of hybrid Li_3PO_4-$AlPO_4$-$Al(PO_3)_3$ coating layer on $LiNi_{0.8}Co_{0.1}Mn_{0.1}O_2$ cathode with enhanced electrochemical properties for lithium-ion battery. *Chem. Eng. J.* **2020**, *382*, 122959.
2. Chen, S.; He, T.; Su, Y.; Lu, Y.; Bao, L.; Chen, L.; Zhang, Q.; Wang, J.; Chen, R.; Wu, F. Ni-rich $Ni_{0.8}Co_{0.1}Mn_{0.1}O_2$ oxide coated by dual-conductive layers as high-performance cathode material for lithium ion batteries. *ACS Appl. Mater. Interfaces* **2017**, *35*, 29732–29743.
3. Dai, S.; Yan, G.; Wang, L.; Luo, L.; Li, Y.; Yang, Y.; Liu, H.; Liu, Y.; Yuan, M. Enhanced electrochemical performance and thermal properties of Ni-rich $LiNi_{0.8}Co_{0.1}Mn_{0.1}O_2$ cathode material via CaF_2 coating. *J. Electroanal. Chem.* **2019**, *847*, 113197.
